# Urogenital cultures and preterm birth in women with cervical cerclage: a single center retrospective cohort study

**DOI:** 10.1186/s12884-024-06509-9

**Published:** 2024-04-26

**Authors:** Evelien Seys, Ann-Sophie Page, Jan Deprest, Lore Lannoo, Kristel van Calsteren, Roland Devlieger, Johannes van der Merwe

**Affiliations:** 1 Department of Obstetrics and Gynecology, Division Woman and Child, University Hospitals Leuven, Herestraat 49, Leuven, 3000 Belgium; 2 Cluster Woman and Child, Department of Development and Regeneration, Group Biomedical Sciences, KU Leuven Herestraat 49, Leuven, 3000 Belgium

**Keywords:** Preterm birth, Cervical cerclage, Urine culture, Vaginal culture

## Abstract

**Background:**

The leading hypothesis of the pathogenesis of cervical insufficiency suggests a role of cervical inflammation. Urogenital tract infections could play a causative role in this process. To test this hypothesis in women with a cervical cerclage, we aimed to retrospectively examine the relationship between gestational age (GA) at delivery and positive urogenital cultures.

**Methods:**

This single center retrospective study reviewed the records of all women with a singleton pregnancy that underwent cervical cerclage (*n* = 203) between 2010 and 2020 at the University Hospital of Leuven, Belgium. Transvaginal cerclages were categorized as history indicated (TVC I, *n* = 94), ultrasound indicated (TVC II, *n* = 79) and clinically indicated (TVC III, *n* = 20). Additionally, ten women received transabdominal cerclage (TAC). Urogenital cultures (vaginal and urine) were taken before and after cerclage with 4-week intervals. Urogenital cultures were reported ‘positive’ if urine and/or vaginal cultures showed significant growth of a microorganism. Treatment decision depended on culture growth and clinical presentation. The primary aim was to evaluate the association between the urogenital culture results and the GA at delivery, for each of the cerclage groups. Secondarily, to investigate the effect of antibiotic treatment of positive cultures on GA at delivery.

**Results:**

Positive pre-cerclage urogenital cultures were associated with lower GA at delivery in TVC III (positive culture 26w4d ± 40d vs. negative 29w6d ± 54d, *p* = 0.036). For TVC I, GA at delivery was longer when pre-cerclage urogenital cultures were positive (positive culture 38w0d ± 26d vs. negative 35w4d ± 42d, *p* = 0.035). Overall post-cerclage urogenital cultures status was not associated with a different GA at delivery. Treating patients with pre- or post-cerclage positive urogenital cultures did also not change GA at delivery.

**Conclusion:**

Positive urogenital cultures taken before clinically indicated cerclage intervention may be associated with lower GA at delivery. However, there seems to be no benefit of antibiotic treatment or routine urogenital cultures during follow-up of asymptomatic women after cerclage placement.

**Supplementary Information:**

The online version contains supplementary material available at 10.1186/s12884-024-06509-9.

## Background

Worldwide roughly 1 out of 10 children are born preterm, i.e. before 37 weeks of gestation [1]. Extreme preterm birth (PTB) (< 28 weeks) leads to perinatal death in 53% of cases in Belgium [2]. Children that survive extreme PTB have a pertinent higher risk of cognitive and motor development problems, attention disorders and social difficulties compared with those born after 28 weeks [3]. Hence, gestational age (GA) at delivery is the most determining factor of all neonatal outcomes.

Cervical insufficiency, defined as the inability of the cervix to retain the pregnancy, causes 8–43% of mid-trimester losses [4, 5] and 11–65% of all spontaneous preterm births (sPTB) [6–8]. It can be due to previous direct trauma, for example caused by cervical loop electrosurgical excision or conization or it can be a consequence of a congenital malformation such as a Müllerian anomaly. When cervical insufficiency exists in absence of these causes, the pathogenesis remains unclear. At present, the leading hypothesis suggests a role of cervical inflammation caused by lower urogenital tract infections [9]. Indeed, pyelonephritis, if left untreated, is a known risk factor for sPTB, and in the last decade an association was also found with lower urinary tract infections. Even asymptomatic bacteriuria in pregnant women is recommended to be treated to reduce the risk of pyelonephritis and therefore reduce the risk of sPTB; hence at our institution it is part of routine screening. Furthermore, concerning genital infections, bacterial vaginosis (BV) in pregnancy is recognized as a strong predictor of sPTB. Moreover, some authors speculate that Candida, Chlamydia and Gonorrhea could be associated with sPTB [10]. Therefore urogenital tract infections could lead to cervical insufficiency, and if they remain untreated they may ascend into the uterine cavity [10, 11]. In fact, microbial invasion of the amniotic cavity is present in 51% of women with cervical insufficiency [12].

Cervical cerclage is the only technique to effectively treat cervical insufficiency and ultimately decrease the risk of late miscarriage and extreme PTB [13, 14]. Cervical cerclage is recommended for women with either (1) recurrent spontaneous late miscarriages i.e. history indicated; (2) cervical shortening (< 25 mm) in high risk women undergoing ultrasound surveillance/screening i.e. ultrasound indicated; or (3) painless midtrimester cervical dilatation with exposed fetal membranes i.e. clinically indicated. Cervical cerclage is usually applied via a transvaginal approach, while a transabdominal approach, prior to or early in pregnancy, is only considered in women with a previously failed transvaginal cerclage or when placement of transvaginal cerclage is technically impossible [15]. In literature, the effect of cervical cerclage on the prolongation of pregnancy and increase of neonatal survival is most evident for clinically indicated, i.e. emergency or rescue, cerclage. However, this type of cerclage reports a higher risk of intraoperative rupture of membranes and when an intra-amniotic infection is present, the positive effect on prolonging the pregnancy is decreased [16].

At present, it remains unclear how women with cervical cerclage should be followed-up during pregnancy. The role of routine urogenital culture screening in asymptomatic women with a cerclage remains unproven, even more specifically it is unclear if the treatment of these cultures reduces the risk of sPTB. The primary aim of this study was to examine the relationship between the presence of microorganisms found in pre- or post-cerclage vaginal or urine cultures (i.e. positive cultures) and GA at delivery in women who underwent cervical cerclage depending on their cervical cerclage indication. In line with the leading hypothesis that ascending urogenital infections may lead to sPTB in women with cervical insufficiency [10–12], we hypothesized that positive urogenital cultures would negatively impact GA at delivery in women who underwent cervical cerclage. The secondary aim of this study was to investigate the effect of antibiotic treatment on GA at delivery in women with positive urogenital cultures depending on the cervical cerclage indication. We hypothesized that treatment of positive urogenital cultures would have positive impact on GA at delivery. Thirdly, we conducted an exploratory analysis on a range of other maternal and neonatal outcomes and their relationship with positive urogenital cultures and the effect of antibiotic treatment depending on the cervical cerclage indication. Finally, we added a narrative description of the cases with pre- or post-cerclage vaginal cultures positive for Gardnerella vaginalis (GV), given that a meta-analysis of 2013 has reported that BV doubles the risk of preterm birth [10]. Ultimately, the results of this study may guide local and international recommendations for routine assessment of urogenital cultures in patients with cervical cerclage as was practiced at our institution.

## Methods

### Patients and setting

This single center retrospective cohort study reviewed the records of all women with singleton pregnancies who underwent cervical cerclage (transvaginal or transabdominal) between 01-01-2010 and 05-12-2020 at the University Hospital of Leuven, Belgium and was approved by the local Ethical Committee (reference: MP017199). Over this period of 10 years, one woman could have more than one pregnancy in which a cerclage was placed. In that case, this woman was included in our study as many times as her number of pregnancies with cerclage. The medical history of this women was described in correspondence with the situation at the time of the respective cerclage.

Data was collected from medical files, including maternal demographics and baseline obstetric and gynecologic characteristics with antenatal and postnatal information. The primary endpoint was GA at delivery. Secondary endpoints included cerclage specific (all factors related to the cerclage procedure, for example antibiotics and tocolysis used during procedure, the suture used for the cerclage, complications due to the procedure etc.), maternal morbidity and neonatal outcomes. Additional file 1 shows the list of variables that was used for data retrieval.

### Categorization of cervical cerclage indications

All included pregnancies were categorized in 4 groups: (1) history-indicated transvaginal cerclage (TVC I), (2) ultrasound-indicated transvaginal cerclage (TVC II), (3) clinically-indicated transvaginal cerclage (TVC III) and (4) transabdominal cerclage (TAC) placed either antenatal or prenatal.

### Local clinical and surgical protocol

According to standardized clinical protocol at our institution: (1) All women were followed every 2 weeks in a dedicated preterm birth clinic, starting from 16 weeks of gestation, with a transvaginal ultrasound for cervical length assessment. (2) Mid-stream urine and cervicovaginal cultures were taken with 4-week intervals, or shorter in symptomatic patients or when deemed clinically necessary. PCR analysis was only performed when Chlamydia, Neisseria, Mycoplasma or Ureaplasma species were suspected by clinical presentation of the patient. Urogenital culture was found ‘positive’ if urine and/or vaginal culture showed significant growth of a specific bacteria or mycosis. Treatment was dependent on culture growth and clinical presentation. Pre-cerclage culture refers to the last culture taken before the procedure and post-cerclage refers to any culture taken during the course of the pregnancy afterwards. Furthermore, we described if positive cultures were found to be de novo, recurrent (repeated culture of same organism after successful treatment) or persistent (repeated culture of same organism despite treatment). (3) All patients were advised to take vaginal progesterone 200 mg daily (*n* = 203). (4) In case of uncomplicated TVC, cervical cerclages were electively removed between 36 and 37 weeks of gestation and in case of TAC, women underwent caesarean section at 37 weeks of gestation.

The local protocol for cervical cerclage placement included preoperative prophylactic antibiotics (cefazolin) and tocolysis (indomethacin), the placement of a monofilament non-braided suture (Ethilon) or a Multifilament braided suture (Mersilene tape) using a McDonald or Shirodkar approach under spinal or general anesthesia. The choice of technique and suture depended on the surgeons’ preference and experience. Transabdominal cerclages were placed by an open abdominal or laparoscopic technique using a Multifilament braided suture (Mersilene tape) under general anesthesia.

### Statistics

Statistical data analysis was performed using IBM SPSS Statistics 27.0 (SPSS Inc, Chicago, IL). Continuous data was checked for normal distribution, defined as kurtosis and skewness between − 2 and + 2, and checked graphically using histograms. Results are reported as means and standard deviations (SD) for continuous variables. Categoric variables are described as frequencies with their percentages. We used one-way ANOVA to compare between the four cerclage indication groups. In case the normality assumption was not met, the Kruskal-Wallis Test was used as a non-parametric alternative (this was the case for pre-cerclage CRP, neutrophils and stitch to delivery interval days [SDI]). Independent samples T-tests were performed for post-hoc analyses when comparing continuous variables between two groups for each cerclage indication. When the normality assumption was not met, the Mann-Whitney U test was used as a non-parametric alternative (this was the case for SDI). For post-hoc analyses of categorical variables we used Pearson’s chi-squared test/Two-sided Fisher’s exact test. Level of significance was defined as α < 0.05.

## Results

### Descriptives and group differences

Between January 2010 and December 2020, 203 cerclage procedures were performed during 202 pregnancies (one case underwent a redo emergency TVC) on 168 women. Table 1 summarizes the baseline characteristics of the patients. The initial preoperative presentation, details on the cerclage placement and general outcomes are given in Table 2. Ninety-four women received a TVC I, 79 women a TVC II, 20 women a TVC III and 10 women a TAC (from which 8 received the TAC in the same pregnancy in an early stage and 2 women already had a TAC in situ from a previous pregnancy). Forty women (21%) received a TVC in two subsequent pregnancies, from which 22 women (14%) received both TVC during the 10 year study period. Nineteen women (10%) underwent TVC in at least three pregnancies, from which 6 women (4%) received three TVC during the study period. From the 10 pregnancies with TAC, four women had one TVC in their history, one woman had one TVC and one TAC in her history, and one woman had already undergone a TAC in a previous pregnancy (before our studied period).

**Table 1:** Baseline characteristics

	History indicated (TVC I, *n* = 94)	Ultrasound indicated (TVC II, *n* = 79)	Clinically indicated (TVC III, *n* = 20)	Abdominal cerclage (TAC, *n* = 10)	Total (*n* = 203)	*p*-value
Maternal age	32.1 ± 4.8	32.0 ± 5.5	31.3 ± 5.6	36.1 ± 4.8	32.2 ± 5.2	0.088
BMI	24.6 ± 4.2	23.8 ± 3.7	25.6 ± 3.4	25.9 ± 4.1	24.5 ± 3.9	0.163
No maternal comorbidity	57 (61)	44 (56)	10 (50)	7 (70)	118 (58)	0.669
Caucasian ethnicity	60 (67)	53 (68)	12 (60)	7 (70)	132 (67)	0.914
African ethnicity	17 (19)	12 (15)	5 (25)	1 (10)	35 (18)	0.673
Education level^a^: high	48 (87)	37 (80)	7 (58)	4 (67)	96 (81)	0.700
Education level^a^: medium	4 (7)	8 (17)	4 (33)	2 (33)	18 (15)	0.058
Education level^a^: low	3 (6)	1 (2)	1 (8)	0 (0)	5 (4)	0.106
Smoking during pregnancy	4 (5)	8 (11)	3 (16)	1 (10)	16 (8)	0.306
Drug use during pregnancy	7 (7)	4 (5)	2 (10)	0 (0)	13 (6)	0.677
Alcohol during pregnancy	1 (1)	0 (0)	1 (5)	0 (0)	2 (1)	0.241
Uterine malformation^b^	2 (2)	5 (6)	0 (0)	0 (0)	7 (3)	0.320
Medical history of						
- LLETZ/conization	12 (13)	18 (23)	1 (5)	6 (60)	36 (18)	0.000*
- Surgical curettage	6 (6)	2 (3)	4 (20)	0 (0)	12 (6)	0.023*
- Miscarriage < 8w	44 (47)	32 (41)	8 (40)	4 (40)	88 (43)	0.839
- Miscarriage 8-15w6d	9 (10)	5 (6)	0 (0)	2 (20)	16 (8)	0.227
- Miscarriage 16-23w6d	66 (70)	21 (27)	5 (25)	8 (80)	100 (49)	0.000*
- PTB 24-27w6d	19 (20)	5 (6)	2 (10)	3 (30)	29 (14)	0.027*
- PTB 28-33w6d	14 (15)	13 (17)	2 (10)	2 (20)	31 (15)	0.876
- PTB 34-36w6d	10 (11)	7 (9)	1 (5)	3 (30)	21 (10)	0.175
Gravidity	4 (1–10)	2 (1–9)	2 (1–8)	4 (2–12)	3 (1–12)	0.000*
Previous CD	26 (28)	24 (30)	4 (20)	3 (30)	57 (28)	0.833
Previous uterine rupture	0 (0)	1 (1)	1 (5)	0 (0)	2 (1)	0.221
Previous TVC 1x	37 (40)	3 (4)	0 (0)	5 (50)	45 (22)	0.000*
Previous TVC > 1	16 (17)	1 (1)	2 (10)	0 (0)	19 (9)	0.003*
Previous TAC only	0 (0)	2 (3)	0 (0)	1 (10)	3 (2)	0.063

*p < 0.05. Categoric parameters are expressed as n and (%) of total within group. Continuous parameters are expressed as mean ± SD, except for Gravidity in median and range. Abbreviations: BMI, body mass index; PTB, preterm birth; LLETZ, large loop excision of transformation zone; CD, caesarean delivery; TVC, transvaginal cerclage; TAC, transabdominal cerclage. ^a^Education level ‘high’ - patient went to university or higher education, ‘medium’ - patient finished secondary education level, ‘low’ - patient did not finish secondary education level. ^b^Uterine malformations include: septate uterus, hemi-uterus; bicorporeal uterus, uterus didelphys

**Table 2:** Pre-operative presentation, cerclage placement and general outcomes

	History indicated (TVC I, *n* = 94)	Ultrasound indicated (TVC II, *n* = 79)	Clinically indicated (TVC III, *n* = 20)	Abdominal cerclage (*n* = 10)	*p*-value	
**Pre-operative**						
Asymptomatic presentation	90 (96)	57 (72)	14 (70)	10 (100)	0.000*	
Presentation with vaginal bleeding	3 (3)	2 (3)	4 (20)	0 (0)	0.005*	
Pres. with abdominal discomfort	0 (0)	20 (25)	3 (15)	0 (0)	0.000*	
TVU cxl (mm)	36.0 ± 7.4	14.9 ± 7.1	0.0 ± 0.0	23.1 ± 6.8	0.000*	
TVU funnel length (mm)	0.1 ± 0.9	10.5 ± 10.9	25.9 ± 11.8	0 ± 0.0	0.000*	
TVU sludge	1 (1)	25 (33)	12 (63)	0 (0)	0.000*	
CRP (mg/L)	8.3 ± 8.3	6.1 ± 7.7	6.5 ± 5.3	NA	0.194	
leucocytes (10*9/L)	9.3 ± 2.6	9.9 ± 2.3	10.2 ± 2.7	8.8 ± 2.4	0.500	
neutrophils (10*9/L)	11.9 ± 8.7	7.5 ± 2.2	7.2 ± 2.4	6.2 ± 2.3	0.300	
**Cerclage placement**						
GA (days)	96.0 ± 8.7	138.4 ± 23.5	147.5 ± 15.1	92.9 ± 5.6^a^	0.000*	
Multifilament/braided suture	29 (39)	29 (39)	8 (47)	10 (100)	0.003*	
Monofilament/PDS suture	46 (61)	36 (55)	9 (53)	0 (0)	0.003*	
Peroperative antibiotics	61 (68)	57 (76)	17 (85)	9 (90)	0.209	
Peroperative tocolysis	50 (56)	53 (69)	14 (74)	8 (80)	0.143	
General anesthesia	9 (11)	5 (7)	2 (12)	10 (100)	0.000*	
Spinal anesthesia	54 (67)	50 (69)	12 (71)	0 (0)	0.000*	
Epidural anesthesia	18 (22)	18 (25)	3 (18)	0 (0)	0.342	
Peroperative complications	0 (0.0)	0 (0)	0 (0)	0 (0)	NA	
**Maternal and neonatal outcomes**						
Postoperative CxL (mm)	33.9 ± 8.6	25.0 ± 8.4	17.5 ± 9.9	30.8 ± 6.3	0.000*	
Postoperative complications	9 (10)	2 (3)	4 (20)	1 (10)	0.055	
Displacement	3 (3)	2 (3)	1 (5)	1 (10)	0.648	
PROM	11 (12)	14 (18)	5 (25)	0 (0)	0.200	
GA at delivery (weeks and days)	36w1d ± 40d	35w2d ± 39d	28w3d ± 49d	36w6d ± 3d	0.000*	
SDI (days)	144.7 ± 34.3	98.1 ± 38.1	47.9 ± 47.9	NA	0.000*	
PTB < 37w	26 (28)	33 (42)	16 (84)	4 (40)	0.000*	
PTB < 34w	20 (22)	18 (23)	14 (74)	0 (0)	0.000*	
PTB < 32w	14 (15)	16 (21)	12 (63)	0 (0)	0.000*	
PTB < 28w	10 (11)	10 (13)	11 (58)	0 (0)	0.000*	
PTB < 24w	7 (8)	6 (8)	7 (35)	0 (0)	0.000*	
Birthweight (g)^b^	2970 ± 940	2687 ± 918	1622 ± 1211	2793 ± 366	0.000*	
Early neonatal death^c^	0 (0)	0 (0)	4 (20)	0 (0)	0.000*	
Alive at discharge	87 (93)	71 (90)	8 (42)	10 (100)	0.000*	
No neonatal complications	56 (60)	39 (50)	4 (21)	5 (5)	0.022*	

*p < 0.05. Categoric parameters are expressed as n and (%) of total within group. Continuous parameters are expressed as mean ± SD. ^a^Mean GA of transabdominal cerclage placement during pregnancy, as 2 were placed pregestational. ^b^Birthweight for life born fetuses. ^c^Early Neonatal Death: life born fetuses who die before 8th day after birth. Abbreviations: GA, gestational age; Cxl, cervical length; PROM, premature rupture of membranes; SDI, stitch delivery interval days; TVU, transvaginal ultrasound; NA, not applicable

No intraoperative complications occurred (rupture of membranes, cervix laceration or other). However, during the procedure a significant cervical lesion was noted in 3 cases which affected the placement and efficacy of the cerclage. The first case had a visible defect on the cervix from a previous cerclage. Her cerclage spontaneously ruptured at 18 weeks followed by a premature rupture of membranes (PROM) at 28 weeks gestational age. The second case had a fistula from the exocervical surface to the endocervical canal from a previous cerclage. The suture was placed behind the fistula. This second case had a PROM at 25 weeks gestational age. In the third case, the medical file reported a lacerated cervix with a shortened portio vaginalis of 5 mm, leading to spontaneous erosion of the cerclage. All three cases delivered preterm (between 30 and 34 weeks of gestation).

Postoperative complications occurred in 8.9% of cases (18/203). The postoperative complications were displacement (*n* = 7), redo emergency transvaginal cerclage (*n* = 1), cerclage knot failure with subsequent removal of transvaginal cerclage (*n* = 3), cerclage erosion through the intracervical canal (*n* = 2), cervix laceration during removal of the cerclage (*n* = 1), and failed removal of transvaginal cerclage (*n* = 4).

Pre-cerclage cultures were positive in 7% (15/203) of urine cultures (TVC I 5/94, 5%; TVC II 8/79, 10%; TVC III 1/20, 5%; TAC 1/10, 10%; *p* = 0.632) and in 26% (52/203) of vaginal cultures (TVC I 19/94, 20%; TVC II 23/79, 29%; TVC III 8/20, 40%; TAC 2/10, 20%; *p* = 0.232). Microbiological results from urine cultures showed cases with Enterococcus faecalis, Escherichia coli and Streptococcus agalactiae. Microbiological results from vaginal cultures showed cases with Streptococcus agalactiae, Gardnerella vaginalis, Proteus mirabilis, Escherichia coli, Candida albicans, Candida glabrata, Candida lusitaniae, Staphylococcus aureus and Candida parapsilosis. See Additional file 2 for the specific incidence results per microbe by cerclage group. Only the incidence of Candida lusitaniae was significantly different between the four groups as it was only found once in the TAC group (*p* = 0.000). Vaginal cultures were positive with Gardnerella vaginalis in 21% of the pregnancies in TVC I (4/94), 22% in TVC II (5/79), 13% in TVC III (1/20) and none in TAC (0/10) (*p* = 0.851).

The incidence and results of post-cerclage cultures are given in Table 3. Post-cerclage cultures were positive in 13% (26/203) of urine cultures (TVC I 11/94, 13%; TVC II 12/79, 15%; TVC III 2/20, 10%; TAC 1/10, 10%; *p* = 0.762) and 39% (80/203) of vaginal cultures (TVC I 37/94, 45%; TVC II 31/79, 46%; TVC III 8/20, 40%; TAC 4/10, 40%; *p* = 0.965).

**Table 3:** Specific culture results and incidence of post-cerclage cultures

	History indicated (TVC I, *n* = 94)	Ultrasound indicated (TVC II, *n* = 79)	Clinically indicated (TVC III, *n* = 20)	Abdominal cerclage (*n* = 10)	*p*-value
Positive urine culture	11 (13)	12 (15)	2 (10)	1 (10)	0.762
- Recurrent	2 (2)	3 (4)	0 (0)	0 (0)	0.685
- Persistent	1 (1)	3 (4)	0 (0)	0 (0)	0.477
Urine micro-organisms					
- Streptococcus agalactiae	5 (45)	4 (33)	0 (0)	0 (0)	0.475
- Staphylococcus aureus	2 (18)	0 (0)	0 (0)	0 (0)	0.406
- Proteus mirabilis	2 (18)	0 (0)	0 (0)	0 (0)	0.406
- Escherichia coli	2 (18)	4 (33)	2 (100)	0 (0)	0.097
- Enterococcus faecalis	0 (0)	2 (17)	0 (0)	1 (100)	0.181
- Citrobacter koseri	0 (0)	1 (8)	0 (0)	0 (0)	0.763
-Staphylococcus haemolyticus	0 (0)	2 (17)	0 (0)	0 (0)	0.480
- Klebsiella pneumoniae	1 (9)	2 (17)	0 (0)	0 (0)	0.846
- Streptococcus anginosus	0 (0)	1 (8)	0 (0)	0 (0)	0.763
Positive vaginal culture^a^	37 (45)	31 (46)	8 (40)	4 (40)	0.965
- New onset	20 (21)	15 (19)	5 (25)	3 (30)	0.856
- Recurrent	8 (10)	8 (12)	2 (13)	0 (0)	0.696
- Persistent	9 (11)	8 (12)	1 (6)	1 (10)	0.937
Vaginal micro-organisms					
- Streptococcus agalactiae	11 (30)	7 (23)	0 (0)	0 (0)	0.210
- Proteus mirabilis	3 (8)	1 (3)	0 (0)	0 (0)	0.674
- Klebsiella pneumoniae	4 (11)	4 (13)	0 (0)	0 (0)	0.661
- Klebsiella oxytoca	1 (3)	0 (0)	0 (0)	0 (0)	0.769
- Gardnerella vaginalis	9 (24)	8 (26)	3 (38)	1 (25)	0.902
- Escherichia coli	8 (22)	5 (16)	3 (38)	1 (25)	0.631
- Enterococcus faecalis	3 (8)	2 (7)	0 (0)	0 (0)	0.806
- Citrobacter koseri	0 (0)	1 (3)	0 (0)	0 (0)	0.672
- Candida lusitania	0 (0)	1 (3)	0 (0)	0 (0)	0.672
- Candida glabrata	0 (0)	3 (10)	0 (0)	0 (0)	0.182
- Candida albicans	15 (41)	10 (32)	4 (50)	1 (25)	0.738
- Staphylococcus aureus	3 (8)	1 (3)	0 (0)	0 (0)	0.674
- Serratia marcescens	0 (0)	1 (3)	0 (0)	0 (0)	0.672
- Enterobacter cloacae	0 (0)	1 (3)	0 (0)	0 (0)	0.672
- Ureaplasma	1 (3)	0 (0)	0 (0)	0 (0)	0.769
Positive other cultures	1 (1)	1 (1)	2 (11)	0 (0)	0.068
Antibiotic treatment	32 (40)	28 (38)	12 (60)	4 (40)	0.344
- Multiple treatments	8 (9)	3 (4)	0 (0)	0 (0)	0.301

*p < 0.05. Categoric parameters are expressed as n and (%) of total within group. Continuous parameters are expressed as mean ± SD. ^a^Recurrent: repeated culture of same organism after successful treatment. Persistent: repeated culture of same organism despite treatment. Multiple treatments were given when the specific bacteria did not respond to the primary given antibiotic

### The relationship between urogenital culture results and GA at delivery

Our primary outcome, GA at delivery, was negatively influenced by positive pre-cerclage urogenital cultures in patients with TVC III (pos culture 185 ± 40d vs. neg 209 ± 54d, *p* = 0.036). In contrast, for TVC I GA at delivery was positively influenced by positive pre-cerclage urogenital cultures (pos culture 267 ± 26d vs. neg 249 ± 42d, *p* = 0.021), see also Table 4. Positive post-cerclage cultures did not significantly affect the GA at delivery, see also Table 5.

**Table 4:** Pre-cerclage urogenital cultures

	History indicated (TVC I, *n* = 94)		Ultrasound indicated (TVC II, *n* = 79)		Clinically indicated (TVC III, *n* = 20)	
PRE-CERCLAGE cultures	Positive (21)	Negative (73)	Positive (28)	Negative (51)	Positive (9)	Negative (11)
Last cxl preoperative (mm)	35.2 ± 7.5	36.2 ± 7.4	14.8 ± 6.3	14.9 ± 7.6	0.0	0.0
Postop complications	1 (5)	2 (3)	2 (7)	0 (0)	3 (33)	1 (9)
PROM	1 (5)	10 (14)	6 (21)	8 (16)	2 (22)	9 (82)
GA at delivery (days)	266.8 ± 25.7	248.9 ± 42.3 0.021*	240.9 ± 44.3	250.3 ± 36.1	184.9 ± 40.2	209.5 ± 54.4 0.036*
SDI (days)	146.9 ± 28.6	144.0 ± 36.1	89.6 ± 36.0	102.3 ± 38.7	22.6 ± 38.3	64.1 ± 47.9 0.035*
PTB < 34w	2 (10)	18 (25)	8 (29)	10 (20)	7 (78)	7 (64)
PTB < 28w	1 (5)	9 (12)	4 (14)	6 (12)	6 (67)	5 (46)
PTB < 24w	0 (0)	7 (10)	3 (11)	3 (6)	4 (44)	3 (27)
Neonatal death	0 (0)	0 (0)	0 (0)	0 (0)	1 (11)	2 (18)
Alive at discharge	21 (100)	60 (90)	24 (86)	47 (92)	3 (33)	5 (46)
Birthweight (g)^a^	3248 ± 825	2879 ± 95	2732 ± 728	2662 ± 990	1051 ± 926	2383 ± 118
No neonatal complications	18 (86)	38 (52) 0.002*	12 (43)	28 (55)	1 (11)	3 (27)
PRE-CERCLAGE positive cultures	Treated (10)	Not treated (11)	Treated (21)	Not treated (6)	Treated (5)	Not treated (4)
Last cxl preoperative (mm)	33.9 ± 6.1	36.0 ± 8.8	15.4 ± 5.5	10.2 ± 5.6 0.048*	0.0	0.0
Postop complications	1 (10)	0 (0)	1 (5)	1 (17)	2 (40)	1 (25)
PROM	1 (10)	0 (0)	5 (24)	1 (17)	2 (40)	0 (0)
GA at delivery (days)	257.1 ± 34.7	275.2 ± 8.	239.9 ± 45.3	244.8 ± 48.8	194.8 ± 49.3	175.0 ± 33
SDI (days)	137.2 ± 39.3	156.9 ± 8.5	89.5 ± 31.3	89.8 ± 55.	36.5 ± 48.1	4.0 ± 3.0
PTB < 34w	2 (20)	0 (0)	7 (33)	1 (17)	3 (60)	4 (100)
PTB < 28w	1 (10)	0 (0)	3 (14)	1 (17)	3 (60)	3 (75)
PTB < 24w	0 (0)	0 (0)	2 (10)	1 (17)	1 (20)	3 (75)
Neonatal death	0 (0)	0 (0)	1 (5)	0 (0)	1 (20)	0 (0)
Alive at discharge	10 (100)	11 (100)	18 (86)	5 (83)	3 (60)	1 (25)
Birthweight (g)^a^	2957 ± 1049	3482 ± 568	2756 ± 765	2683 ± 751	1313 ± 1239	790 ± 533
No neonatal complications	7 (70)	11 (100)	10 (48)	2 (33)	1 (20)	0 (0)

Categoric parameters are expressed as n and (%) of total within group. Continuous parameters are expressed as mean ± SD. *p < 0.05, only significant p-values are shown for each variable indicated in the second column for each indication group. ^a^Birthweight for life born fetuses. Abbreviations: Cxl, cervical length; GA, gestational age; PROM, premature rupture of membranes; SDI, stitch delivery interval days

**Table 5:** Post-cerclage urogenital cultures

	History indicated (TVC I, *n* = 94)		Ultrasound indicated (TVC II, *n* = 79)		Clinically indicated (TVC III, *n* = 20)	
POST-CERCLAGE cultures	Positive (39)	Negative (44)	Positive (34)	Negative (35)	Positive (10)	Negative (8)
Postop cxl (mm)	34.2 ± 9.0	34.2 ± 8.6	24.9 ± 8.4	24.6 ± 8.8	18.5 ± 7.2	17.5 ± 13.5
Postop complications	2 (5)	1 (2)	1 (3)	1 (3)	2 (20)	2 (25)
PROM	6 (15)	5 (11)	3 (9)	10 (29) 0.036*	0 (0)	4 (50) 0.023*
GA at delivery (days)	246.7 ± 42.1	254.9 ± 41.1	253.3 ± 30.8	238.1 ± 45.1	212.3 ± 59.1	192.0 ± 41.2
SDI (days)	141.9 ± 36.7	145.4 ± 34.2	105.8 ± 32.7	91.2 ± 39.7	66.4 ± 49.2	37.4 ± 43.8
PTB < 34w	11 (28)	8 (18)	6 (18)	11 (31)	5 (50)	7 (88)
PTB < 28w	6 (15)	4 (9)	2 (6)	7 (20)	4 (40)	5 (63)
PTB < 24w	3 (8)	4 (9)	1 (3)	4 (11)	3 (30)	3 (38)
Neonatal death	0 (0)	0 (0)	0 (0)	0 (0)	1 (10)	2 (25)
Alive at discharge	36 (92)	40 (91)	33 (97)	29 (83)	5 (50)	3 (38)
Birthweight (g)^a^	2836 ± 1060	3021 ± 875	2746 ± 713	2539 ± 1078	2344 ± 1468	1396 ± 954
No neonatal complications	23 (59)	29 (66)	19 (56)	16 (46)	3 (30)	1 (13)
POST-CERCLAGE positive cultures	Treated (31)	Not treated (5)	Treated (25)	Not treated (9)	Treated (8)	Not treated (2)
Postop cxl (mm)	34.4 ± 9.4	33.8 ± 7.8	25.4 ± 8.8	23.7 ± 7.3	18.6 ± 7.8	18.0 (NA)
Postop complications	2 (7)	0 (0)	1 (4)	0 (0)	1 (13)	1 (50)
PROM	5 (16)	0 (0)	3 (12)	0 (0)	0 (0)	0 (0)
GA at delivery (days)	251.2 ± 39.9	251.2 ± 40.3	250.3 ± 33.4	261.6 ± 21.7	210.4 ± 59.6	219.0 ± 80.
SDI (days)	143.4 ± 37.6	130.25 ± 29.6	102.9 ± 32.4	113.3 ± 34.2	66.7 ± 45.7	65.5 ± 82.7
PTB < 34w	7 (23)	1 (20)	5 (20)	1 (11)	4 (50)	1 (50)
PTB < 28w	4 (13)	1 (20)	2 (8)	0 (0)	3 (38)	1 (50)
PTB < 24w	2 (7)	0 (0)	1 (4)	0 (0)	2 (25)	1 (50)
Neonatal death	0 (0)	0 (0)	0 (0)	0 (0)	1 (13)	0 (0)
Alive at discharge	29 (94)	5 (100)	24 (96)	9 (100)	4 (50)	1 (50)
Birthweight (g)^a^	2853 ± 1073	2728 ± 1086	2665 ± 746	2954 ± 609	2507 ± 1346	2100 ± 2192
No neonatal complications	19 (61)	4 (80)	12 (48)	7 (78)	2 (25)	1 (50)

Categoric parameters are expressed as n and (%) of total within group. Continuous parameters are expressed as mean ± SD. *p < 0.05, only significant p-values are shown for each variable indicated in the second column for each indication group. ^a^Birthweight for life born fetuses. Abbreviations: Cxl, cervical length; GA, gestational age; PROM, premature rupture of membranes; SDI, stitch delivery interval days

Secondarily, we investigated the effect of treating patients with positive urogenital cultures and found that antibiotic treatment did not significantly influence GA at delivery, neither before nor after cervical cerclage.

### The relationship between urogenital culture results and maternal and neonatal outcomes following cervical cerclage

Maternal and neonatal outcomes by pre-cerclage and post-cerclage culture status are given in Tables 4 and 5, respectively. Positive pre-cerclage cultures were associated with a shorter SDI only in TVC III (pos culture 23 ± 38d vs. neg 64 ± 47d, *p* = 0.035) and fewer neonatal complications in TVC I (pos culture 86% vs. neg culture 52% with no neonatal complications, *p* = 0.002). Positive post-cerclage urogenital cultures were associated with fewer ruptures of membranes in TVC II (pos culture 3/34, 9% vs. neg 10/35, 29%; *p* = 0.036) and TVC III (pos culture 0/10, 0% vs. neg 4/8, 50%; *p* = 0.023).

Antibiotic treatment for positive pre-cerclage urogenital cultures was associated with a longer preoperative cervical length (treated 15.4 ± 5.5 mm vs. untreated 10.2 ± 5.6 mm, *p* = 0.048). There were no significant associations between outcome measures and antibiotic treatment for positive post-cerclage urogenital cultures.

Antibiotic treatment in the pre-cerclage setting was given to respectively 12% of TVC I group (11/94), 28% of TVC II (22/79), 25% of TVC III (5/20) and 20% of TAC (2/10). Treatment was depending on culture growth and clinical presentation, and included miconazole (17/44), clindamycin (8/44), amoxicillin (7/44), benzylpenicillin (4/44), amoxicillin-clavulanic acid (3/44), cefuroxime (1/44), clarithromycin 1/44), erythromycin (1/44), nitrofurantoin (1/44) and metronidazole (1/44).

Antibiotic treatment for positive post-cerclage cultures was given to 79% of TVC I cases (31/39), 74% of TVC II (25/34), 80% of TVC III (8/10) and 80% of TAC (4/5). Post-cerclage antibiotics included clindamycin (28/68), miconazole (24/68), amoxicillin-clavulanic acid (21/68), amoxicillin (8/68), cefuroxime (8/68), penicillin (5/68), fosfomycin (4/68), erythromycin (3/68), cefadroxil (2/68), piperacillin-tazobactam (2/68), ampicillin (1/68), nitrofurantoin (1/68), metronidazole (1/68), gentamicin (1/68), vancomycin (1/68), butoconazole nitrate (1/68), nifurtoïnol (1/68).

### The effect of Gardnerella vaginalis infection on mean GA at delivery

Pre-cerclage, 21% of the vaginal cultures were positive with GV in TVC I (4/19, mean GA at delivery 39w4d, SDI 7d), 22% in TVC II (5/23, mean GA at delivery 33w4d, SDI 61d), 13% in TVC III (1/8, GA at delivery 22w2d, SDI 10d) and none in TAC (0/2) (*p* = 0.851). At post-cerclage, 24% of the vaginal cultures were positive with GV in TVC I (9/37, mean GA at delivery 36w1d, SDI 40d), 26% in TVC II (8/31, mean GA at delivery 37w4d, SDI 6d), 38% in TVC III (3/8, mean GA at delivery 38w1d, SDI 10d) and 25% in TAC (1/4, GA at delivery 37w2d) (*p* = 0.902). Three cases maintained GV from pre-cerclage to post-cerclage (TVC I, GA at delivery 40w1d, SDI 163d; TVC I, GA at delivery 39w4d, SDI 156d; and TVC II, GA at delivery 37w0d, SDI 105d).

In 4 out of 31 cases positive with GV, delivery was before 36 weeks of gestational age. Three cases had a late miscarriage due to chorioamnionitis. 4 out of 31 cases positive with GV were left untreated, however, without impact on their GA at delivery as they all delivered at term. Detailed description can be found in Additional file 3.

## Discussion

This retrospective observational study aimed to investigate the relationship between pre- or post-cerclage urogenital cultures and GA at delivery in a contemporary cohort of women who underwent cervical cerclage. In line with our hypothesis, positive pre-cerclage cultures in clinically-indicated cerclage negatively influenced GA at delivery (*p* = 0.036). However, positive pre-cerclage urogenital cultures in history-indicated group had a positively influence on the GA at delivery (*p* = 0.021). Additionally, treating positive cultures did not significantly affect GA at delivery. The latter results should be interpreted with care, because of a small number of patients in some of the groups.

Our main findings are in line with two previous studies who reported that positive vaginal culture at time of clinically indicated cerclage is an independent risk factor for PTB [17, 18]. These two studies did also not find an association between post-cerclage positive vaginal cultures and GA at delivery, nor with treated abnormal vaginal flora and GA at delivery. Compared to our results, Gundabuttulaet et al. reported a higher incidence of positive pre-cerclage vaginal (64%) and urine (15%) cultures [17]. In this study, the cultured organisms on vaginal swab were mostly *Escherichia coli* (14%), followed by *Klebsiella* (10%), *Enterococcus* (2%), *Staphyloccous aureus* (2%) [17]. In the second study, Kanbayashi et al. reported mainly *GV* (30%), followed by *Group B streptococcus* (25%) and *Staphyloccocus* species (8.3%) in pre-cerclage vaginal cultures [18]. In contrast, we mainly cultured *Streptococcus agalactiae* (25%) and *GV* (13%) on the vaginal swab before clinically indicated cerclage. The heterogeneity between cultured micro-organisms from vaginal cultures in association with PTB suggest that multiple organisms could have an impact on PTB in clinically-indicated cerclage. Hence, we advise routine pre-cerclage culture tests and treatment of positive cultures in those undergoing a clinically-indicated cerclage.

Other studies report contrasting results to ours. For example, Jin et al., reported that women with an ultrasound-indicated transvaginal cerclage and *Escherichia coli* in post-cerclage vaginal cultures had a higher risk of PTB < 37 weeks (*p* = 0.023) [19]. Furthermore, Kunpalin et al. investigated a cohort of 251 women with either a history or ultrasound indicated cerclage. They found that positive pre-cerclage urine cultures were associated with a significantly lower GA at delivery (36.6 vs. 38.3 weeks; *p* = 0.040), while no such relationship was found for vaginal cultures, or urogenital post-cerclage cultures [20]. The reason for these contrasting results could be that Kunpalin et al. mainly cultured *Escherichia coli* (47%) in pre-cerclage urine cultures, while in our series *Enterococcus* species was the most common urinary pathogen (40–50%). In contrast to *Escherichia coli*, *Enterococcus* species are mainly reflecting contamination or colonization and are rarely associated with perinatal outcomes [21]. With regards to vaginal cultures, Kunpalin et al. reported pre-cerclage growth of BV in 20% of women with a history or ultrasound indicated cerclage, similar to our findings. The incidence of positive vaginal cultures post-cerclage in our study (TVC I 45%, TVC II 46%) was higher than reported by Kunpalin et al. (24%) [20] and was three times higher than the incidence of positive post-cerclage urine cultures.

Importantly, only one previous study reported on the incidence of positive urogenital cultures in women with TAC. Pre-cerclage, Akeno et al. reported a GV infection in 21% of cases, while no pre-cerclage GV infections were found in our TAC group [22]. Post-cerclage, Akeno et al. reported no GV infections [22], while we had one case with GV (25%) found in post-cerclage vaginal culture in the TAC group. This finding in our cohort did not seem to have implications on cerclage outcome.

Facultative bacteria, such as GV, or anaerobic bacteria such as *Mobiluncus* species, or *Mycoplasma* species such as *Ureaplasma urealyticum*, that overgrow in the normal vaginal flora cause BV, a well-described risk factor for PTB [23, 24]. A meta-analysis of 2013 showed that the risk of preterm birth was doubled when BV was found; and even seven times higher if it occurred before 16w [25]. However treatment of this disbalance has not yet been proven to be able to reduce the risk of preterm birth in asymptomatic women [10]. In our study, of all pregnancies with GV positive cultures pre- or post-cerclage, only four women (13%) delivered before 36 weeks of gestation. Our results did not suggest a negative effect of GV on the mean GA at delivery nor on the mean SDI in any of the cerclage indication subgroups. In line with our findings, a Cochrane review of 2013 did not suggest any benefit in screening and treating pregnant women for asymptomatic BV to prevent preterm birth [26].

We should note that in the clinically indicated TVC III group, three patients presented with abdominal discomfort. At the time of presentation these patients did not have signs of contraction on cardiotocogram and were clinically not considered in labor after thorough questioning and examination. Therefore, the discomfort was not categorized as labour or suggestive of chorioamnionitis. Furthermore, cervical insufficiency is defined by the American College of Obstetricians and Gynecologists (ACOG) as the inability of the uterine cervix to retain a pregnancy in the second trimester, in the absence of uterine contractions. It typically presents as an acute, painless dilatation of the cervix, which at a later stage evolves into a status with evident non-stoppable uterine contractions leading to premature delivery [7]. Within the group of women presenting with abdominal discomfort in the clinically indicated subgroup, a SDI below 30 days was noted in two of them, specifically 1 and 6 days. Moreover, also three women with ultrasound-indicated cervical cerclage who presented with abdominal discomfort had a SDI below 30 days, specifically 6, 16 and 23 days. However, this highlights the ambiguity that could exists when treating these patients and the reason why some authors suggest a time of observation before a decision is made to place a clinically indicated cerclage [27].

The main strength of this study is the detailed description of outcomes after cervical cerclage in a large cohort of women that were managed according to a single institutional protocol. Urogenital cultures were routinely performed in all women. This study is the first to report these outcomes in Belgian women, which could aid local, regional and national institutions to review their guidelines. However, due to the retrospective design, this study has some inherent limitations. Foremost, our study can only report associations, without making claims on causality. Furthermore, we were not always able to describe the full clinical context of all cultures, as well as the reason for the chosen antibiotic regime. Finally, the small number of patients in some of the subgroups, especially in the number of patients undergoing antibiotic treatment in each of the cerclage indication subgroups, limits our ability to make general conclusions.

## Conclusions

Consistent with previous studies, we did not observe an association between the presence of positive pre-cerclage urogenital cultures and GA at delivery in any of the cerclage indication groups, except for clinically-indicated (rescue) transvaginal cerclage. Furthermore, there tends to be no benefit of routine use of urogenital cultures during follow-up of asymptomatic women after cerclage placement. Antibiotic treatment of patients with positive pre- or post-cerclage urogenital cultures did also not impact GA at delivery in our study cohort.

### Electronic supplementary material

Below is the link to the electronic supplementary material.

Supplementary Material 1

Supplementary Material 2

Supplementary Material 3

## Data Availability

The dataset used and analysed during the current study is available from the corresponding author on reasonable request.

## Abbreviations

BV: bacterial vaginosis
GA: gestational age
GV: Gardnerella vaginalis
PROM: Premature rupture of membranes
PTB: Preterm birth
SD: Standard deviation
sPTB: spontaneous preterm birth
TAC: Transabdominal cerclage
TVC: Transvaginal cerclage
TVC I: History indicated transvaginal cerclage
TVC II: Ultrasound indicated transvaginal cerclage
TVC III: Clinically indicated transvaginal cerclage

## References

[1] World Health Organization. WHO recommendations on interventions to improve preterm birth outcomes. 2015. https://www.who.int/publications/i/item/9789241508988. Accessed 12 March 2023. 26447264

[2] Devlieger R, Goemaes R, Laubach M. Perinatale gezondheid in Vlaanderen – Jaar 2020. Brussel: Studiecentrum voor Perinatale Epidemiologie. 2021; 1–85.

[3] Morgan AS, Mendonça M, Thiele N, David AL (2022). Management and outcomes of extreme preterm birth. BMJ.

[4] Drakeley AJ, Quenby S, Farquharson RG (1998). Mid-trimester loss - Appraisal of a screening protocol. Hum Reprod.

[5] Joubert M, Sibiude J, Bounan S, Mandelbrot L (2022). Mid-trimester miscarriage and subsequent pregnancy outcomes: the role of cervical insufficiency in a cohort of 175 cases. J Matern Neonatal Med.

[6] Lidegaard O (1994). Cervical incompetence and cerclage in Denmark 1980–1990 a register based epidemiological survey. Acta Obstet Gynecol Scand.

[7] Barinov SV, Artymuk NV, Novikova ON, Shamina IV, Tirskaya YI, Belinina AA (2021). Analysis of risk factors and predictors of pregnancy loss and strategies for the management of cervical insufficiency in pregnant women at a high risk of preterm birth. J Matern Neonatal Med.

[8] Vink J, Feltovich H (2016). Cervical etiology of spontaneous preterm birth. Semin Fetal Neonatal Med.

[9] Roman A, Suhag A, Berghella V (2016). Overview of cervical insufficiency: diagnosis, etiologies, and risk factors. Clin Obstet Gynecol.

[10] Cunnington M, Kortsalioudaki C, Heath P (2013). Genitourinary pathogens and preterm birth. Curr Opin Infect Dis.

[11] Obert R, Oldenberg LG, Ohn J, Auth CH, Ndrews WA (2000). Intrauterine infection and Preterm Delivery. N Engl J Med.

[12] Digiulio DB, Gervasi M, Romero R, Vaisbuch E, Mazaki-Tovi S, Kusanovic JP (2010). Microbial invasion of the amniotic cavity in pregnancies with small-for-gestational-age fetuses. J Perinat Med.

[13] Care A, Nevitt SJ, Medley N, Donegan S, Goodfellow L, Hampson L (2022). Interventions to prevent spontaneous preterm birth in women with singleton pregnancy who are at high risk: systematic review and network Meta-analysis. Obstet Gynecol Surv.

[14] Alfirevic Z, Stampalija T, Medley N (2017). Cervical stitch (cerclage) for preventing preterm birth in singleton pregnancy. Cochrane Database Syst Reviews.

[15] Shennan A, Chandiramani M, Bennett P, David AL, Girling J, Ridout A et al. MAVRIC: a multicenter randomized controlled trial of transabdominal vs transvaginal cervical cerclage. Am J Obstet Gynecol. 2020;222(3):261.e1-261.e9. 10.1016/j.ajog.2019.09.040 31585096

[16] Chatzakis C, Efthymiou A, Sotiriadis A, Makrydimas G (2020). Emergency cerclage in singleton pregnancies with painless cervical dilatation: a meta-analysis. Acta Obstet Gynecol Scand.

[17] Gundabattula SR, Marakani LR, Dasari S, Surampudi K, Pochiraju M, Nirmalan PK (2013). Outcomes of pregnancy in women who had rescue cerclage for cervical insufficiency: a single-center retrospective study. J Obstet Gynaecol Res.

[18] Kanbayashi S, Sato Y, Taga A, Satake Y, Emoto I, Maruyama S (2018). Positive vaginal culture at rescue cerclage predicts subsequent preterm delivery. J maternal-fetal Neonatal Med.

[19] Jin W, Hughes K, Sim S, Shemer S, Sheehan PM (2022). Abnormal vaginal flora and spontaneous preterm birth in high-risk singleton pregnancies with cervical cerclage. J maternal-fetal Neonatal Med.

[20] Kunpalin Y, Burul G, Greenwold N, Tetteh A, Casagrandi D, Warner D (2020). Factors associated with preterm birth in women undergoing cervical cerclage. Eur J Obstet Gynecol Reproductive Biology.

[21] Schnarr J, Smaill F (2008). Asymptomatic bacteriuria and symptomatic urinary tract infections in pregnancy. Eur J Clin Invest.

[22] Akeno K, Ohashi M, Furukawa S, Sameshima H (2022). Cerclage in surgically shortened uterine cervix and pregnancy outcome: a retrospective comparison between the abdominal and vaginal procedures. J Obstet Gynaecol Res.

[23] Cunnington M, Kortsalioudaki C, Heath P (2013). Genitourinary pathogens and preterm birth. Curr Opin Infect Dis.

[24] Nasioudis D, Linhares IM, Ledger WJ, Witkin SS (2017). Bacterial vaginosis: a critical analysis of current knowledge. BJOG: Int J Obstet Gynecol.

[25] Leitich H, Bodner-Adler B, Brunbauer M, Kaider A, Egarter C, Husslein P (2003). Bacterial vaginosis as a risk factor for preterm delivery: a meta-analysis. Am J Obstet Gynecol.

[26] Brocklehurst P, Gordon A, Heatley E, Milan SJ. Antibiotics for treating bacterial vaginosis in pregnancy. Cochrane Database Syst Rev. 2013. 10.1002/14651858.CD000262.pub4 PMC11307253 23440777

[27] Gómez-Castellano M, Sabonet-Morente L, González-Mesa E, Jiménez-López JS (2022). A Three-Step Procedure for Emergency Cerclage: gestational and neonatal outcomes. Int J Environ Res Public Health.

