# Supplementary material for: Urogenital cultures and preterm birth in women with cervical cerclage: a single center retrospective cohort study
Source: BMC Pregnancy Childbirth. 2024 Apr 26;24:324. doi: 10.1186/s12884-024-06509-9 (PMC11046802; doi:10.1186/s12884-024-06509-9)
Supplement: Supplementary file 1 — Supplementary Material 1 [file 12884_2024_6509_MOESM1_ESM.docx]

Additional file 1: list of variables used for data retrieval

| 1. Women’s general health | |
| --- | --- |
| 1.1 Date of birth | dd/mm/yyyy |
| 1.2 UZ Leuven EAD | xxxxxxxxxxx |
| 1.3 Maternal Age | Auto Field |
| 1.4 Ethnicity | 1. Caucasian; 2. African (incl Moroccan and Congolese); 3. Turkish; 4. Arabic; 5. Asian; 6. Chinese; 7. Hispanic; 8. Other; 9. Unknown |
| 1.5 Education Level | 1. High; 2. Medium; 3. Low; 4. Not specified |
| 1.6 Occupation | 0. Unemployed; 1. Part Time; 2. Full time; 3. Not specified |
| 1.7 Weight (kg) | xx |
| 1.8 Height (cm) | xx |
| 1.9 BMI | Auto Field |
| 1.10 Smoking | 0. Never; 1. Stopped before pregnancy; 2. Stopped during pregnancy; 3. Smoking <5/day; 4. Smoking >5/day |
| 1.11 Social Drug Use | 0.Never; 1. Alcohol; 2. Cannabis; 3. Methamphetamine; 6. Other |

| 1. Women’s medical history | |
| --- | --- |
| 2.1 Medical Conditions pre-existing | 0. None, 1. essential hypertension, 2. cardiac disease (congenital or acquired), 3. renal disease, 4. endocrine disorders e.g. hypo or hyperthyroidism, 5. psychiatric disorders, 6. hematological disorders e.g. sickle cell disease, 7. diagnosed thrombophilia, 8. inflammatory disorders e.g. inflammatory bowel disease, 9. epilepsy, 10. Diabetes, 11. autoimmune diseases, 12. cancer, 13. HIV, 14. fibromateuze uterus, 15. other, please specify, 16. unknown, 17. bariatric surgery |
| 2.2 Medication use before pregnancy (free text) |  |
| 2.3 Uterine anomalies | 0. normal uterus, 1. subseptate uterus, 2. septate uterus, 3. unicornuate uterus, 4. bicornuate uterus, 5. didelphys uterus |
| 2.4 Cervical surgery | 2.4.1 LLETZ  0. No or Number xx  2.4.2 Conization  0. No or Number xx  2.4.3 Trachelectomy  0. No or 1. Yes |
| 2.5 Gynaecology Procedures | \| 2.5.1. Vacuum aspiration (curettage)  0. No or Number xx \| \| --- \| \| 2.5.2. Surgical Curettage  0. No or Number xx \| \| 2.5.3. Myomectomy  0. No, 1. Laparotomy, 2. Laparoscopic \| \| 2.5.4. Hysteroscopy  0. No, 1. Polyp resection, 2. Septum resection, 3. Fibroid resection, 4. Endometrial ablation, 5. Placental tissue removal, 6. Asherman \| \| 2.5.5. Previous uterine perforation  0. No, 1. During curettage, 2. During myomectomy, 3. During hysteroscopy \| \| 2.5.6. Previous uterine rupture  0. No, 1. Yes, 2. Unknown \| \| 2.5.7. Previous fetal surgery  0. No, 1. Laser, 2. RFA, 3. Fetoscopy (CDH), 4. Open (MMC), 5. Lsc MMC, 6. Transfusions, 7. Other \| |

| 1. Previous pregnancies | |
| --- | --- |
| Gravidity | xx |
| Parity >24w | 0-xx |
| Alive | 0-xx |
| Miscarriage <8w | 0-xx |
| Miscarriage 8-15w6d | 0-xx |
| Miscarriage 16-23w6d | 0-xx |
| PTB 24-27w6d | 0-xx |
| PTB 28-33w6d | 0-xx |
| PTB 34-36w6d | 0-xx |
| Previous induced miscarriage | 0-xx |
| Previous induced labour | 0-xx |
| Previous vaginal delivery | 0-xx |
| Previous cesarean delivery | Auto Field |
| Previous elective cesarean delivery | 0-xx |
| Previous emergency cesarean delivery | 0-xx |
| Previous sPTB (24-36w6d) | 0-xx |
| Earliest sPTB GA xxw | xx |
| Earliest sPTB GA xd | x |
| Earliest sPTB GA calc | Auto Field |
| Previous cerclage | 0.no, 1. vaginal 1x, 2. vaginal >1, 3. vaginal and abdominal, 4. abdominal only |
| Last cerclage GA xxw | xx |
| Last cerclage GA xxd | xx |

| 1. This pregnancy | |
| --- | --- |
| EDD | (dd/mm/yyyy) |
| Dating US date | (dd/mm/yyyy) |
| GA date US (w) | xx |
| GA date US (d) | x |
| GA date US Calculated | Auto Field |
| Cervical length screening | 0. No; 1. At 20w US; 2. From 14-16w |
| CxL Screening from GA xxw | xx |
| CxL Screening from GA xd | x |
| CxL Screening from GA Calculated | Auto Field |
| Clinical Presentation | 0. None; 1. PVB/antepartum hemorrhage; 2. abdominal pressure/pain; 3. other (specify) |
| Vag Bleeding | 0. No, 1. Limited single episode; 2. Limited multiple episodes; 3. Complicated heavy bleed with admission |
| Vag Bleeding GA xxw | xx |
| Vag Bleeding GA xd | x |
| Vag Bleeding GA cal | Auto Field |
| TVUCxL last/preop (mm) | xx |
| TVU Funnel Length (mm) | xx |
| TVU Funnel Width (mm) | xx |
| TVU Sludge | 0 None; 1 Present |
| Clinical dilatation (mm) | 0-xx |

| 1. Cerclage in this pregnancy | |
| --- | --- |
| Precerclage Urine Culture/PCR | 0. Not done; 1. Neg; 2. Pos Culture |
| Precerclage Urine Microbe |  |
| Precerclage Vaginal Culture/PCR | 0. Not done; 1. Neg; 2. Pos Culture |
| Precerclage Vaginal Microbe |  |
| Precerclage other Cultures/PCR | 0. Not done; 1. Neg; 2. Pos Culture |
| Precerclage other Microbe |  |
| Precerclage Amnio Culture/PCR | 0. Not done; 1. Neg; 2. Pos Culture |
| Precerclage Amnio Microbe |  |
| Precerclage culture Rx | 0. No; 1. Yes; 3. Not specified |
| Precerclage antibiotics |  |
| Amnio Genetic Analysis | 0. Not done; 1. Neg; 2. Pos |
| Genetic Abnormality (free tekst) |  |
| Precerclage CRP (mg/L) | xxx |
| Precerclage Leucocyte (10*9/L) | xxx |
| Precerclage Neutrophil (10*9/L) | xxx |
| Cerclage indication | 1. History based preventative; 2. Ultrasound indicated; 3. Clinically indicated |
| Cerclage Type | 1. VC McD; 2. VC Sch; 3. TAC open; 4. TAC Lsc |
| Cerclage Timing | 1. During pregnancy; 2. Prepregnancy/interval |
| Cerclage Suture | 0. Not specified; 1. Multifilament/braided (Mersilene); 2. Monofilament PDS |
| Cerclage Date | (dd/mm/yyyy) |
| Cerclage Date GA xxw | xx |
| Cerclage Date GA xd | x |
| Cerclage Date GA cal | Auto Field |
| Anesthesia | 0 Nil; 1 Spinal; 2 Epidural; 3 GA |
| Peroperative antibiotics | 0. No; 1. yes |
| Peroperative Tocolyse | 0. No; 1. NSAIDs; 2. CaBlockers; 3. Atociban |
| Cerclage complications Intra-operative | 0 Nil; 1 ROM; 2 Laceration; 3 Other |
| Cerclage bleeding intra-operative (mL) | xxx |
| Cerclage complications Post-Operative | 0 Nil; 2 Displacement; 3 ROM; 4 revision; 5 Infection; 6 other (specify) |

| 1. Post cerclage | |
| --- | --- |
| 1st Postop Cerclage Diameter (mm) | xx |
| 1st Postop Cervixlength (mm) | xx |
| 1st Postop Cervixlength above cerclage (mm) | xx |
| Last Postop Cerclage Diameter (mm) | xx |
| Last Postop Cervixlength (mm) | xx |
| Last Postop Cervixlength above cerclage (mm) | xx |
| Postcerclage Urine Culture/PCR | 0. Not done; 1. Neg; 2. Single Pos; 3. Persistent Pos; 4. Recurrent Pos |
| Postcerclage Urine Microbes |  |
| Postcerclage Vaginal Culture/PCR | 0. Not done; 1. Neg; 2. Single Pos; 3. Persistent Pos; 4. Recurrent Pos |
| Postcerclage Vaginal Microbes |  |
| Postcerclage Other Culture/PCR | 0. Not done; 1. Neg; 2. Single Pos; 3. Persistent Pos; 4. Recurrent Pos |
| Postcerclage Other Microbes |  |
| Postcerclage culture Rx | 0. No; 1. Single course; 2. Multiple Courses |
| Postcerclage Antibiotics |  |
| Pregnancy Complications | 0. None, 1. pre‐eclampsia (hypertension and proteinuria), 2. Eclampsia, 3. thrombotic event, 4. amniotic fluid embolism, 5. miscarriages, 6. preterm birth or mid trimester loss, 7. Stillbirth, 8. baby with a major congenital abnormality, 9. small for gestational age (SGA) infant, 10. large for gestational age (LGA) infant, 14. gestational diabetes, 15. significant placental abruption, 19. ruptured uterus, 20. PROM (premature rupture of membranes), 21. TOP after foeticide, 22. TOP without foeticide, 23. other (please specify), 24. unknown |
| Problems (Free Tekst) |  |

| 1. End of pregnancy | |
| --- | --- |
| Antenatal Admission | 0-xx |
| Antenatal Corticosteroids Timing | 0. No; 1. Within 7d of delivery; 2. >7d from delivery |
| Antenatal Corticosteroids Courses | 0. No; 1. Single course; 2. Multiple Courses |
| Antenatal Corticosteroids 1st GA w | xx |
| Antenatal Corticosteroids 1st GA d | x |
| Tocolysis | 0. No; 1. Single course; 2. Multiple Courses |
| MgSO4 | 0. No; 1. Single course; 2. Multiple Courses |
| Stitch Remova date | (dd/mm/yyyy) |
| Delivery Date | (dd/mm/yyyy) |
| GA Delivery (w) | xx |
| GA Delivery (d) | x |
| GA Delivery Calculate Days | Auto Field |
| GA Delivery Calc Days Check | Auto Field |
| PTB <37w | 0. >37w; 1. < 37w; 2. <34w; 3. < 32w; 4. < 28w; 5. <24w |
| Stitch Interval (days) | Auto Field |
| Labour Onset | 0. None; 1. Spontaneous onset; 2. IOL with prostaglandins; 3. IOL Mechanical; 4. IOL AROM |
| Delivery Mode | 1. Spontaneous vaginal, 2. Assisted vaginal, 3. Elective CD, 4. CD other |
| Pregnancy Outcome | 1. Miscarriage <24w; 2. Stillbirth >24w; 3. Neonatal death; 4. Alive at discharge |
| Placental Pathology | 0. Not done; 1. No pathology noted; 2. Chorioamnionitis; 3. Chorioamnionitis + Funisitis; 4. Ischemic placental disease; 5. Abruptio; 6. Other |
| Culture cerclage | Yes, No |
| Results culture cerclage (free text) |  |

| 1. Neonatal period | |
| --- | --- |
| Birthweight (g) | 0-xxx |
| Sex | 1. Male, 2. Female, 3. Ambiguous, 4. Unknown |
| Apgar 5 | x |
| UA pH | x.xx |
| UV pH | x.xx |
| NICU/HDU Admission (days) | 0-xx |
| Neonatal Complications | 0. None, 1. Respiratory distress syndrome, 2. Intraventricular hemorrhage, 3. Necrotizing enterocolitis, 4. Neonatal encephalopathy, 5. Chronic lung disease, 6. Severe jaundice requiring phototherapy, 7. Major congenital anomaly, 8. Severe infection e.g. septicemia, meningitis, 9. Exchange transfusion, 10. Neonatal death, 11. Other |
| Neonatal Mortality Date | (dd/mm/yyyy) |
| Neonatal Mortality Cause (free text) |  |
| Neonate EAD | xxxxxxxxx |
| Follow up > 2y | Yes No |
