# Supplementary material for: Urogenital cultures and preterm birth in women with cervical cerclage: a single center retrospective cohort study
Source: BMC Pregnancy Childbirth. 2024 Apr 26;24:324. doi: 10.1186/s12884-024-06509-9 (PMC11046802; doi:10.1186/s12884-024-06509-9)
Supplement: Supplementary file 2 — Supplementary Material 2 [file 12884_2024_6509_MOESM2_ESM.docx]

**Additional file 2**

| Additional file 2: Specific culture results and incidences of pre-cerclage cultures | | | | | |
| --- | --- | --- | --- | --- | --- |
|  | History indicated  (TVC I, n=94) | Ultrasound indicated  (TVC II, n=79) | Clinically indicated  (TVC III, n=20) | Abdominal cerclage  (n = 10) | p-value |
| Positive urine culture | 5 (5) | 8 (10) | 1 (5) | 1 (10) | 0.632 |
| - Enterococcus faecalis  - Escherichia coli  - Streptococcus agalactiae | 2 (40)  1 (20)  2 (40) | 4 (50)  1 (13)  2 (25) | 1 (100)  0 (0)  0 (0) | 1 (100)  0 (0)  0 (0) | 0.622  0.942  0.813 |
| Positive vaginal culture | 19 (20) | 23 (29) | 8 (40) | 2 (20) | 0.232 |
| - Streptococcus agalactiae  - Gardnerella vaginalis  - Proteus mirabilis  - Escherichia coli  - Candida albicans  - Candida glabrata | 3 (16)  4 (21)  0 (0)  0 (0)  10 (53)  1 (5) | 8 (35)  5 (22)  1 (4)  1 (4)  11 (48)  2 (8) | 2 (25)  1 (13)  0 (0)  0 (0)  4 (50)  1 (13) | 0 (0)  0 (0)  0 (0)  0 (0)  1 (50)  0 (0) | 0.460  0.851  0.750  0.750  0.993  0.901 |
| -Candida lusitaniae | 0 (0) | 0 (0) | 0 (0) | 1 (50) | 0.000* |
| -Staphylococcus aureus | 0 (0) | 1 (4) | 0 (0) | 0 (0) | 0.669 |
| -Candida parapsilosis | 1 (5) | 0 (0) | 0 (0) | 0 (0) | 0.765 |
| -Candida non-albicans | 1 (5) | 0 (0) | 0 (0) | 0 (0) | 0.765 |
| Antibiotic treatment | 11 (12) | 22 (28) | 5 (25) | 2 (20) | 0.068 |
| *p<0.05. Categoric parameters are expressed as n and (%) of total within group. Continuous parameters are expressed as mean ± SD. | | | | | |
