# Supplementary material for: Urogenital cultures and preterm birth in women with cervical cerclage: a single center retrospective cohort study
Source: BMC Pregnancy Childbirth. 2024 Apr 26;24:324. doi: 10.1186/s12884-024-06509-9 (PMC11046802; doi:10.1186/s12884-024-06509-9)
Supplement: Supplementary file 3 — Supplementary Material 3 [file 12884_2024_6509_MOESM3_ESM.docx]

**Additional file 3**

Two cases with GV positive pre-cerclage vaginal cultures delivered before 36 weeks gestational age, one from the TVC II (GA at delivery 20w6d, SDI 2d) and one from the TVC III (GA at delivery 22w2d, SDI 10d) subgroup and two cases with GV positive post-cerclage cultures delivered before 36 weeks gestational age, both from the TVC I subgroup (GA at delivery 34w2d, SDI 136d; and GA at delivery 21w3d, SDI 66d). A detailed description of the three cases who delivered before 24w of gestation (i.e. late miscarriage) can be found below. All three cases developed chorioamnionitis.

GV was treated in our center with clindamycin vaginal for 7 days. Four cases with GV were left untreated, two with positive pre-cerclage (TVC I, GA at delivery 40w1d, SDI 168d; and TVC II, GA at delivery 37w0d, SDI 105d) and two with positive post-cerclage cultures (TVC I, GA at delivery 38w3d, SDI 161d; and TVC III, GA at delivery 39w3d, SDI 124d).

*Case 1: Gardnerella vaginalis cultured in pre-cerclage vaginal swab in TVC II group.*

The first case was a 31 year old, Asian, primigravida. She had a high educational level and was full-time employed. She did not smoke and did not use drugs or alcohol. Her BMI was 28. She had a normal uterus and no medical maternal history. She presented asymptomatic for an ultrasound indicated cerclage. Upon presentation, there was sludge visible. Pre-cerclage urine culture was negative, while vaginal culture showed GV. She underwent transvaginal cerclage at 20w4d of gestation. The McDonald technique was used with a monofilament suture under spinal anesthesia with intra-operative antibiotics and tocolysis. No intraoperative nor postoperative complications occurred. Postoperative cervical length was 25mm. At 20w6d of gestation, she was diagnosed with premature rupture of membranes with evolving chorioamnionitis. At first, amoxicillin-clavulanic acid was given. However, soon after, it was decided to start medical induction of labor with prostaglandin.

*Case 2: Gardnerella vaginalis cultured in pre-cerclage vaginal swab in TVC III group.*

The second case was a 31 years old, African, primigravida. She had a low educational level and was unemployed. She did not smoke, did not use drugs, but was known with alcohol abuse. Her BMI was 28. She had a fibromatous uterus with normal cavity and was HIV positive (treated). She presented asymptomatic for a clinically indicated cerclage. Pre-cerclage urine culture was negative, while vaginal culture showed GV. Clindamycin was prescribed pre-cerclage. She underwent transvaginal cerclage at 20w2d of gestation. The McDonald technique was used with a multifilament suture under spinal anesthesia with intraoperative antibiotics and tocolysis. No intraoperative nor postoperative complications occurred. Postoperative cervical length was 7mm. Urine and vaginal cultures were negative in the post-cerclage period. At 21w3d, she was diagnosed with premature rupture of membranes, and amoxicillin-clavulanic acid and erythromycin were started with the aim to prevent chorioamnionitis. Subsequently, the cervical suture was removed (SDI of 10d) and conservative therapy was decided. Two days later she left the hospital against medical advice. She returned after two days in premature labor and delivered via spontaneous vaginal delivery at 22w2d of gestational age. Diagnosis of chorioamnionitis was confirmed post-hoc on placental culture.

*Case 3: Gardnerella vaginalis cultured in post-cerclage vaginal swab in TVC I group.*

The third case was a 27 year old, Arabic women with three previous pregnancies that had all ended with miscarriages (one at 8w GA and two between 16 and 23w6d GA). She had a high educational level and was full-time employed. She did not smoke and did not use drugs or alcohol. Her BMI was 34. She had a normal uterus and had undergone a transvaginal cerclage in one of her previous pregnancies at 21w0d GA. She presented asymptomatic for a history indicated cerclage. Pre-cerclage urine and vaginal cultures were negative. She underwent transvaginal cerclage at 12w0d of gestation. The McDonald technique was used with a multifilament suture under spinal anesthesia with no intraoperative antibiotics nor tocolysis. No intraoperative nor postoperative complications occurred. Postoperative cervical length was 33mm. Post-cerclage urine cultures were negative, while post-cerclage vaginal cultures showed recurrent infection with GV despite a single treatment course with clindamycin. Her NIPT test was inconclusive for trisomy 18. At 21w3d (SDI 66d) she presented with premature labor and delivered via spontaneous vaginal. Diagnosis of chorioamnionitis was confirmed post-hoc on placental culture.
